# The mzIdentML Data Standard Version 1.2, Supporting Advances in Proteome Informatics*[Fn FN2]

**DOI:** 10.1074/mcp.M117.068429

**Published:** 2017-05-17

**Authors:** Juan Antonio Vizcaíno, Gerhard Mayer, Simon Perkins, Harald Barsnes, Marc Vaudel, Yasset Perez-Riverol, Tobias Ternent, Julian Uszkoreit, Martin Eisenacher, Lutz Fischer, Juri Rappsilber, Eugen Netz, Mathias Walzer, Oliver Kohlbacher, Alexander Leitner, Robert J. Chalkley, Fawaz Ghali, Salvador Martínez-Bartolomé, Eric W. Deutsch, Andrew R. Jones

**Affiliations:** From the ‡European Molecular Biology Laboratory, European Bioinformatics Institute (EMBL-EBI), Wellcome Trust Genome Campus, Hinxton, Cambridge, CB10 1SD, United Kingdom;; §Medizinisches Proteom Center (MPC), Ruhr-Universität Bochum, D-44801 Bochum, Germany;; ¶Institute of Integrative Biology, University of Liverpool, Liverpool, L69 7ZB, UK;; ‖Proteomics Unit, Department of Biomedicine, University of Bergen, Norway;; **Computational Biology Unit, Department of Informatics, University of Bergen, Norway;; ‡‡KG Jebsen Center for Diabetes Research, Department of Clinical Science, University of Bergen, Norway;; §§Center for Medical Genetics and Molecular Medicine, Haukeland University Hospital, Bergen, Norway;; ¶¶Wellcome Trust Centre for Cell Biology, University of Edinburgh, Edinburgh EH9 3BF, United Kingdom;; ‖‖Chair of Bioanalytics, Institute of Biotechnology Technische Universität Berlin, 13355 Berlin, Germany;; *^a^*Biomolecular Interactions group, Max Planck Institute for Developmental Biology, Tübingen D-72076, Germany;; *^b^*Center for Bioinformatics, University of Tübingen, 72076 Tübingen, Germany;; *^c^*Dept. of Computer Science, University of Tübingen, Germany;; *^d^*Quantitative Biology Center, University of Tübingen, Germany;; *^e^*Department of Biology, Institute of Molecular Systems Biology, ETH Zurich, Auguste-Piccard-Hof 1, 8093 Zurich, Switzerland;; *^f^*Department of Pharmaceutical Chemistry, University of California San Francisco, San Francisco, California, 94143;; *^g^*Department of Chemical Physiology, The Scripps Research Institute, 10550, N. Torrey Pines Rd., La Jolla, California, 92037;; *^h^*Institute for Systems Biology, Seattle, WA, 98109

## Abstract

The first stable version of the Proteomics Standards Initiative mzIdentML open data standard (version 1.1) was published in 2012—capturing the outputs of peptide and protein identification software. In the intervening years, the standard has become well-supported in both commercial and open software, as well as a submission and download format for public repositories. Here we report a new release of mzIdentML (version 1.2) that is required to keep pace with emerging practice in proteome informatics. New features have been added to support: (1) scores associated with localization of modifications on peptides; (2) statistics performed at the level of peptides; (3) identification of cross-linked peptides; and (4) support for proteogenomics approaches. In addition, there is now improved support for the encoding of *de novo* sequencing of peptides, spectral library searches, and protein inference. As a key point, the underlying XML schema has only undergone very minor modifications to simplify as much as possible the transition from version 1.1 to version 1.2 for implementers, but there have been several notable updates to the format specification, implementation guidelines, controlled vocabularies and validation software. mzIdentML 1.2 can be described as backwards compatible, in that reading software designed for mzIdentML 1.1 should function in most cases without adaptation. We anticipate that these developments will provide a continued stable base for software teams working to implement the standard. All the related documentation is accessible at http://www.psidev.info/mzidentml.

The Proteomics Standards Initiative (PSI)^1^ has taken the role of developing standard file formats for different aspects of mass spectrometry (MS) based analysis (for a review see (1)). These include the mzML format, which can store raw MS data suitable for quantitation processes, as well as processed peak lists for searching (2). Downstream of mzML, several formats serve different use cases. To capture the results of identification approaches, such as the use of proteomics search engines, the mzIdentML format was released as a stable version (version 1.1) in the last quarter of 2011, and published in 2012 (3). A separate format for quantitative results was built, called mzQuantML—initially defined for large scale discovery approaches (4) and updated to also support targeted approaches, such as selected reaction monitoring (5). More recently, in 2014, a lighter tab-delimited format called mzTab was also developed by the PSI to capture identification and quantification results (6).

mzML, mzIdentML, and mzQuantML are represented in the Extensible Markup Language (XML). The three formats are defined by an XML Schema Definition (XSD) file, which have all been fixed on a version for some time: mzML (version 1.1 since 2009), mzIdentML (version 1.1 since 2011), and mzQuantML (version 1.0 since 2013). The PSI has also built a large controlled vocabulary (CV), the PSI-MS CV (7), containing more than 2700 terms and definitions in a structured hierarchy (version 4.0.12, May 2017). To ensure that terms are used correctly within the formats, an additional mechanism has been built on top of XSD validation, called PSI semantic validation (8). This mechanism is encoded as a *CV mapping file* listing all places in each format where CV terms are allowed, and specifying a branch or branches of the PSI-MS CV where terms can be sourced, called a *semantic rule*. Each semantic rule is accompanied by a requirement level - MAY, SHOULD or MUST (formal keywords), triggering behavior in custom validation software (information, warning, and error, respectively). To create a valid mzIdentML file thus requires it to be syntactically correct (passing XSD validation) and semantically correct (passing PSI semantic validation *i.e.* intended CV terms are present and correct), and these features have been implemented in validation software (9).

Since its initial stable release, the adoption of mzIdentML 1.1 has increased enormously (Table I). Most notably, several popular proteomics software now export mzIdentML natively, and this list is growing regularly. This includes analysis tools such as MS-GF+ (10), Mascot (*Matrix Science,* London, UK, from version 2.4), ProteinPilot (*SCIEX,* Framingham, MA, from version 5.0), ProteinLynx Global Server (*Waters Corp.*, Milford, MA, from version 3.0.3 onwards), PEAKS (Bioinformatics Solutions, Waterloo, Canada) (11), Scaffold (Proteome Software, Portland, OR) (12), Byonic (*Protein Metrics*), MyriMatch (13), PeptideShaker (14), Crux (15), OpenMS (16), mzID in Bioconductor (17), and PIA (Protein Inference Algorithms) (18). It is also planned that ProteomeDiscoverer (Thermo Fisher Scientific, Waltham, MA) will export the format natively in its next version, to be released later in 2017. It is used as an import format to quantitation software, such as Progenesis QI for proteomics (Waters Corp.) *e.g.* to import results from Byonic. In addition, it is becoming increasingly common that mzIdentML becomes the final output of analysis pipelines, *e.g.* ProteoAnnotator (19). For software not natively implementing the format, some converters are available, *e.g.* ProCon (20) and ProteoWizard (21). Visualization software for mzIdentML files is also now available, most notably the open source PRIDE Inspector tool (22), which was updated in 2016 to fully support mzIdentML, and the ProteoIDViewer (9). Some of these tools are reusing open source libraries tailored to the format such as jmzIdentML (23), mzid Library (9), and the ms-data-core-api (24). Finally, it is important to highlight that mzIdentML is now fully supported as a data submission format, via the “complete” submission mechanism (enabling full search capabilities and visualization of the data), to the PRIDE, MassIVE, and jPOST data repositories, as part of the ProteomeXchange Consortium (25).

**Table I:** A summary of current software available for processing mzIdentML 1.1+ files by May 2017. Tool = Tool name, followed by (Vendor) [Converter if non-native support]. Type = “Search” (Search engine), “Quant” (Quantification software), “IO” (file input/output), “Pipeline” (processing pipeline), “Grouping” (protein grouping), “Post-Processing” (postprocessing routines), “Proteogenomics” (proteogenomics software), “Variant ID” (variant identification software), “Visualization” (visualization tool)—in all cases referring to the named tool. URL = The web address of the tool itself or the conversion utility, if mzIdentML is not natively supported. I/E = IMPORT/EXPORT functionality. F/C = Free/Commercial, F* = the converter is free but the software is not. Additional abbreviations not indicated in the main text: DDA (Data Dependent Acquisition), DIA (Data Independent Acquisition), MRM (Multiple Reaction Monitoring), PRM (Parallel Reaction Monitoring)

Tool	Type	Status/Description	URL	I/E	F/C
Byonic (Protein Metrics Inc.)	Search	Byonic search engine supports mzIdentML 1.1 as an output format	http://www.proteinmetrics.com/products/byonic/	E	C
Crux	Search	Supports mzIdentML 1.1 as an output format and reads mzIdentML 1.1 to generate spectral count data	http://crux.ms/	I & E	F
IDPicker	Grouping	Version 3.x implements mzIdentML 1.1 import	https://medschool.vanderbilt.edu/msrc-bioinformatics/software	I	F
IP2	Search & Quant	*Integrated Proteomics Pipeline* supports export of results into mzIdentML 1.1	http://www.integratedproteomics.com/	E	C
Iquant	Quant	Automated pipeline for quantification by using isobaric tags; identification results are imported *via* mzIdentML 1.1	https://sourceforge.net/projects/iquant/	I	F
jmzIdentML	IO	Java API for reading and writing mzIdentML 1.1	https://github.com/PRIDE-Utilities/jmzIdentML	I & E	F
jPOST	Database	identification result files can be uploaded in mzIdentML 1.1	http://jpostdb.org/	I	F
Mascot (Matrix Science)	Search & Quant	mzIdentML version 1.1 available in Mascot version 2.4+	http://www.matrixscience.com/	E	C
MassIVE	Database	identification files can be uploaded in mzIdentML 1.1	https://massive.ucsd.edu	I	F
ms-data-core-api	IO	Java API that supports reading of PSI standard and open formats e.g. mzML, mzIdentML, mzTab, mgf and others.	https://github.com/PRIDE-Utilities/ms-data-core-api	I	F
MS-GF+	Search	Full support for exporting identification results into mzIdentML 1.1	https://omics.pnl.gov/software/ms-gf	E	F
MyriMatch	Search	Identifications exported in mzIdentML 1.1	https://medschool.vanderbilt.edu/msrc-bioinformatics/software	E	F
mzID package	IO	R package available through Bioconductor supporting v 1.1	http://www.bioconductor.org/packages/release/bioc/html/mzID.html	I	F
mzidLibrary	Post-processing	Routines and viewer (stats, protein inference, CSV import/export, proteogenomics) supporting v1.1 and 1.2	https://github.com/PGB-LIV/mzidlib	I & E	F
OMSSA [mzidLib]	Search	Converter from OMSSA .omx files to v1.1 or 1.2 in mzidLibrary.	https://github.com/PGB-LIV/mzidlib	E	F
OpenMS	Pipeline	mzIdentML 1.1 fully supported in release 1.9 +	https://www.openms.de/	I & E	F
PAnalyzer	Grouping	Used for protein grouping; it imports and exports mzIdentML (v1.1 and 1.2)	https://github.com/akrogp/EhuBio/wiki/Panalyzer	I & E	F
PEAKS (Bioinformatics Solutions Inc.)	Search & Quant	Native export of mzIdentML version 1.1	http://www.bioinfor.com/	E	C
PeptideShaker	Post-processing	Java stand-alone tool for the analysis and post-processing of proteomics experiments; it support mzIdentML 1.1 & 1.2	http://compomics.github.io/projects/peptide-shaker.html	I & E	F
PGA	Proteogenomics	Software for creating RNA-Seq based databases; it supports v1.1 as an input format for post-processing.	http://www.bioconductor.org/packages/devel/bioc/html/PGA.html	I	F
PIA	Grouping	Toolbox for protein inference and identification analysis; it supports mzIdentML 1.1.	https://github.com/mpc-bioinformatics/pia	I & E	F
ProteinLynx Global Server	Search & Quant	Peptide/protein identification and quantification software; it supports export to mzIdentML in version 3.0.3+	www.waters.com/waters/en_GB/ProteinLynx-Global-SERVER-(PLGS)/nav.htm?cid=513821	E	C
PRIDE	Database	mzIdentML 1.1 fully supported as an import format as part of the “complete” dataset submission pipeline	https://www.ebi.ac.uk/pride/archive/	I	F
PRIDE Inspector	Visualisation	Java stand-alone tool that can be used to visualise mzIdentML 1.1 files, independently or together with the corresponding mass spectra files (available in any open formats e.g. mzML, mzXML, mgf, dta, pkl, and apl).	https://github.com/PRIDE-Toolsuite/pride-inspector	I	F
Progenesis QI for proteomics (Waters Corp.)	Quant	Label-free quantification software can read identifications from Byonic in mzIdentML 1.1	http://www.nonlinear.com/progenesis/qi-for-proteomics/	I	C
ProteinPilot	Search & Quant	ProteinPilot 5.0+ exports search results in mzIdentML version 1.2.	https://sciex.com/products/software/proteinpilot-software	E	C
ProteinScape (Bruker)	Search & Quant	It imports search engine results other than Mascot in mzIdentML 1.1	https://www.bruker.com/products/mass-spectrometry-and-separations/ms-software/proteinscape/overview.html	I	C
SEQUEST / Proteome Discoverer (Thermo) [m2Lite / ProCon]	Search & Quant	Conversion of msf files from Proteome Discoverer to mzIdentML 1.1 *via* m2Lite or ProCon (ProCon also supports ProteinScape and Comet conversions).	https://bitbucket.org/paiyetan/m2lite/downloads/	E	F*
			http://www.ruhr-uni-bochum.de/mpc/software/ProCon/index.html.en		
ProteoAnnotator	Proteo-genomics	Proteogenomics software that uses mzIdentML 1.1 as its internal file format	http://www.proteoannotator.org/	E	F
ProteoWizard	IO	pepXML converter available and support for reading/writing mzIdentML 1.1	http://proteowizard.sourceforge.net/	I & E	F
Scaffold	Search & quant	Scaffold 4.0+ supports reading and writing of mzIdentML 1.1	http://www.proteomesoftware.com/products/scaffold/	I & E	C
Skyline	Quant	SRM/MRM/PRM, DIA and targeted DDA software can import mzIdentML 1.1 for spectral library construction	https://skyline.ms	I	F
TagRecon	Variant ID	Identifications exported in mzIdentML 1.1	https://medschool.vanderbilt.edu/msrc-bioinformatics/software	E	F
Trans Proteomic Pipeline [ProteoWizard]	Pipeline	pepXML to mzIdentML 1.1 converter available from ProteoWizard	http://proteowizard.sourceforge.net/	I & E	F
X!Tandem [mzidLib]	Search	Converter from X!Tandem XML files to mzIdentML 1.1 or 1.2 as part of the mzidLibrary.	https://github.com/PGB-LIV/mzidlib	E	F

An important consideration for data standards is the balance between stability and innovation. A standard that is updated at regular intervals causes problems for the developer community—including those writing exporters from their own software, as well as those wishing to write parsers for data produced by others. However, it is also important for standards organisations to update formats periodically as the requirements of the field evolve. In this article, we are reporting an update from mzIdentML version 1.1 to version 1.2 to cope with several features that have been requested by software teams or by the wider proteomics community, and that were not specified previously. New features have generally been implemented by adding new CV terms to the format, and updating the way in which terms are used in a valid mzIdentML file as opposed to making changes to the XML schema itself. However, several minor updates have been made to the mzIdentML XSD file to fix bugs or important omissions (for example concerning whether elements are mandatory or optional), which will overall improve the ease of development around the format. The background to the major new features and improvements is summarized in the following sections. The changes made to mzIdentML 1.2 can be described as backwards compatible, in that reading software designed for mzIdentML 1.1 should function in most cases without adaptation. It is understood that standards should remain stable for significant periods of time to ensure ease of adoption by the developer community, and as such, we have attempted to add new features in a manner that will make it as straightforward as possible for existing adopters of the format.

## 

### 

#### Modification Localization

Even if the identification of a peptide carrying one or more post-translational modifications (PTMs) or chemical modifications can be confirmed with high certainty, the exact residue on which the modification resides may be ambiguous, particularly for modifications that are known to occur on multiple residues, such as phosphorylation on S, T, or Y. A variety of approaches have been developed that give scores or statistical measures to modification localization, as reviewed in (26). There is a growing interest in annotating proteomes with previously “confirmed” PTM sites on proteins, and thus if MS-derived data is to be used for this purpose, it is imperative that the evidence trail is adequately reported in the standard, which could not previously be achieved in a clear way.

#### Cross-linking

Cross-linking MS has become a standard tool in structural biology investigations of multiprotein complexes (27) and can lead to detailed models of proteins (28). The principle of the technique involves the use of a chemical reagent to cross-link residues close in physical space in the three-dimensional structure of a protein or protein complex. Cross-linked peptides are identified by database searching and can reveal which residues are in close proximity in the folded proteins. For instance, such information can be integrated with other sources in structural biology to compute structures of biomolecular systems (29). Upwards of 30 specifically designed search engines and associated statistical techniques are available for identifying cross-linked peptides. The data types resulting from such software require considerable changes to the standard reporting guidelines as used in the rest of the proteomics field.

#### Peptide-level Statistics

In large-scale (discovery) proteomics MS/MS approaches, there has been much work over the last two decades to improve statistical approaches, to ensure that results from different analytical pipelines are more comparable and to accurately estimate the false discovery rate (FDR). The approach as first described was applied to peptide-spectrum matches (PSMs), and the field adopted a consensus 1% FDR threshold. However, high intensity peptides eluting over a retention time higher than the dynamic exclusion of the instrument generate high-similarity fragment spectra which, when correctly identified, result in redundant PSMs, whereas false positives are more evenly distributed across peptides. As a result, if a fixed threshold (*e.g.* 1%) is used for false discovery at the PSM-level, it is likely that the actual level of false discovery at the peptide-level is somewhat higher. This phenomenon is important in a variety of cases, for example the identification of phosphopeptides (or peptides containing other PTMs) or in the annotation of genomes, where it is important to control FDR at the level of the individual peptide sequence. Consequently, new structures have been added to mzIdentML 1.2 to support the grouping of PSMs into peptide units, and the reporting of scores for peptides (as well or instead of PSM scores).

#### Proteogenomics

In these approaches (30), searches are performed against databases that are generated using genomic and/or transcriptomic sequence information, from which novel peptides and sequence variants can be identified. One of the key concepts required is the mapping of peptides back to gene models and chromosomes, for example demonstrating evidence where peptides map across splice junctions. To ensure that a consistent export is possible from mzIdentML to formats designed specifically for genome visualization or annotation, *e.g.* the BED or SAM/BAM formats (31), and their recently developed proteomics counterpart PSI formats proBed (see http://www.psidev.info/probed) and proBAM (http://www.psidev.info/probam), in mzIdentML 1.2, a consistent encoding of the chromosomal mappings for peptides has been developed.

#### Protein Grouping

The protein inference problem has been widely discussed in the literature (32, 33). Most identification pipelines now report grouped protein identifications where ambiguity cannot be resolved *e.g.* proteins have been identified from the same set of peptides. For each group, one or more *leading* or *representative* proteins can be reported. In mzIdentML 1.1, a two-level hierarchy was defined for capturing evidence at the level of the *group*, and the level of individual database *accession numbers*. However, a higher-level concept emerged in some approaches of a protein *cluster* or *family* (34), inside which groups of different proteins shared some peptides in common, but also had independent evidence. The mzIdentML 1.1 specifications left the developers of export software to choose how to use these structures, and the result is that inconsistencies arose around the encoding of clusters/families (which were not explicitly mentioned in the format specification), as well as the definition of group leading proteins. A PSI working group investigated the issue at length, taking on board a wide range of opinions, and examining all popular approaches in software. From the working group a new specification emerged, described previously (35), and now included in this stable release of mzIdentML 1.2.

## EXPERIMENTAL PROCEDURES

The development of mzIdentML 1.2 started in 2012 and it has been an open process *via* conference calls, discussions at the PSI annual meetings and smaller workshops. The specifications have been submitted to the PSI document process (36) for review, during which time external reviewers can provide feedback on the specifications and they are available for public comments, enabling broad input on the specifications. The model is accompanied by CV terms and definitions as part of the PSI-MS CV, also used in other PSI data formats and actively maintained by the PSI MS and PI (Proteomics Informatics) working groups, as well as a newly developed CV for cross-linking reagents and modifications called XLMOD-CV. The complete mzIdentML 1.2 specification document, the new XLMOD-CV, example files and additional documentation can be found at http://www.psidev.info/mzidentml.

### 

#### 

##### mzIdentML Overview

**Fig. 1.**
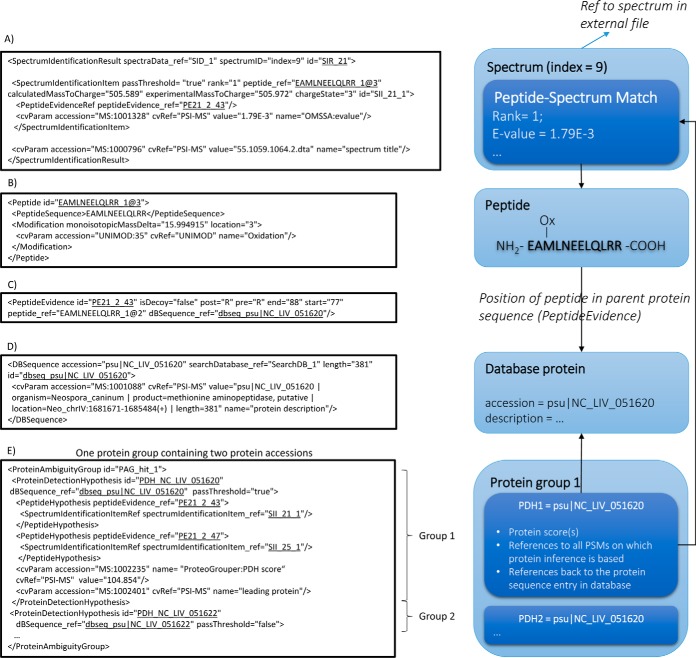
***A*, A single PSM is represented within <*SpectrumIdentificationItem*> including scores as values associated with standard terms sourced from the PSI-MS controlled vocabulary.** Unique identifiers and references to other objects in the file are underlined. *B*, The peptide identified is stored elsewhere in the file, within the <*Peptide*> element, which can be referenced by an unlimited number of PSMs. *C*, All the proteins within which a peptide sequence can be located (given the enzyme specificity as defined) are linked *via* the <*PeptideEvidence*> element. *D*, Database proteins are represented in <*DBSequence*>. *E*, An identified group of proteins is stored within <*ProteinAmbiguityGroup*> (“Protein group” on the right panel) and <*ProteinDetectionHypothesis*> (PDH) - evidence at the level of a single database accession.

Here we briefly describe the structure of mzIdentML files, as a basis for demonstrating the mechanism used to add the new features. As mentioned above, this overall file structure is nearly identical in mzIdentML 1.2 and mzIdentML 1.1. We have attempted to encode the new use cases without changing the core model of the format, to simplify adoption by the developer community. The core model is summarized in Fig. 1. The core data type in proteomics identification approaches (by MS/MS) is the PSM. Most, search engines output one or more ranked explanations (peptide sequences) that match each collected MS/MS fragmentation spectrum, associated with scores or statistical values. In mzIdentML, such data is recorded in a section of the file, called the <*SpectrumIdentificationList*>, which contains a set of elements called<*SpectrumIdentificationResult*>, each storing all reported identifications from a single spectrum. One <*SpectrumIdentificationResult*> has an attribute enabling reading software to identify the spectrum that was searched (in an external file), then lists an ordered set of <*SpectrumIdentificationItem*> elements, each one being a single PSM. The key attributes of the <*SpectrumIdentificationItem*> are the calculated and experimental *m/z* values, the rank, a reference to the peptide that has been identified, and an additional set of scores or statistics, represented as CV terms (list of <*cvParam*> elements). An example PSM represented in mzIdentML (either in version 1.1 or 1.2, the representation does not change) is given in Fig. 1*A*. The <*peptide_ref*> element contains a reference to a separate element in the file containing the <*Peptide*> object, such that if multiple PSMs identify the same peptide, the peptide details are only recorded once in the file to save space (Fig. 1*B*).

The <*SpectrumIdentificationItem*> also has one or more <*PeptideEvidenceRef*> elements (Fig. 1*C*), which reference to a second external object, capturing the protein sequences in which the peptide can be found (assuming a digestion with the given enzyme rules). The <*PeptideEvidence*> element also refers to the <*Peptide*> object and has a second external reference to <*DBSequence*>, which captures a protein sequence entry in the database that was searched (Fig. 1*D*).

Protein and grouped protein results are held in a separate part of the file, called the <*ProteinDetectionList*>. A set of proteins with shared evidence are reported under <*ProteinAmbiguityGroup*>, and the evidence for a single protein accession number being identified is captured under <*ProteinDetectionHypothesis*>, which references the set of PSMs (<*SpectrumIdentificationItem*> elements) on which it is based (Fig. 1*E*). For a complete description of the mzIdentML specification, see the original publication (3) and the PSI website (http://www.psidev.info/mzidentml).

## RESULTS AND DISCUSSION

The following section describes the implementation of new features in mzIdentML 1.2 only. Because of the complexity and diversity of analysis workflows that need to be represented in the mzIdentML 1.2 format, a “flag” was added in the top part of the file, which enables reading software to determine which, if any, new features have been added and need to be considered. This mandatory requirement is met by adding an additional CV term in the <*SpectrumIdentificationProtocol*> element depending on the type of workflow represented (Table II).

**Table II:** New CV terms in the PSI-MS CV that are now mandatory within the element <SpectrumIdentificationProtocol>, enabling the new features in mzIdentML 1.2 to be differentiated and recognized automatically by processing software. In the file, 1 … *n* of the terms MUST be present

CV term name	Accession number	Comments/Purpose
Peptide-level scoring	MS:1002490	Statistics have been performed on non-redundant peptide identifications.
Modification localization scoring	MS:1002491	Scoring has been performed on the sites of peptide modification.
Consensus scoring	MS:1002492	Multiple search engines have been used for peptide identification.
Sample prefractionation	MS:1002493	The file contains the results of merged pre-fractionation analyses.
Cross-linking search	MS:1002494	The search engine has analysed cross-linked (and regular) peptides, using the new encoding described here.
De novo search	MS:1001010	*De novo* sequencing of peptides has been performed, meaning that 0 . . *n* relationships from peptides to proteins are allowed (rather than 1 . . *n*).
Proteogenomics search	MS:1002635	Peptides have been mapped back to genome level coordinates, stored in the file.
Spectral library search	MS:1001031	The identifications have been made by searching against a spectral library. 0 . . *n* peptides to proteins are allowed (rather than 1 . . n elsewhere) for cases where peptide to protein relationships are unknown in the library, or where a library entry has been identified with no known peptide sequence.
No special processing	MS:1002495	Used to indicate that none of the above features have been included in the file.

### 

#### 

##### Modification Position Scoring

**Fig. 2.**
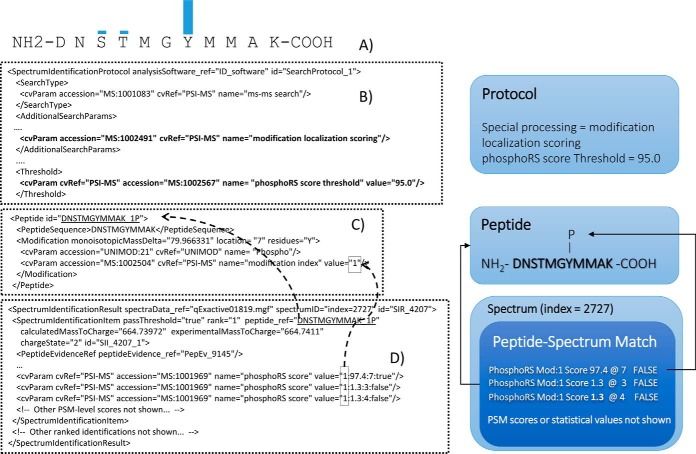
***A*, Graphical representation of the strength of evidence associated with one phosphorylation event on the peptide DNSTMGYMMAK.** *B*, If modification re-scoring has been performed, the protocol must be flagged with the specific CV term and a threshold can be specified as to whether a given modification position has been confidently identified. *C*, The peptide and modification are represented in the re-usable <*Peptide*> and <*Modification*> elements. *D*, Modification localization scores are included within <*SpectrumIdentificationItem*> following a given syntax: *MOD_INDEX:SCORE:POSITION:PASS_THRESHOLD,* where *MOD_INDEX* is the value referenced, allowing different modification types within a given <*Peptide*> element to be referenced, and POSITION is the position along the peptide chain (zero = N terminus; peptide-length + 1 = C terminus).

First, to ensure that downstream software is aware that a file contains modification position scores, a CV term is added to the <*SpectrumIdentificationProtocol*> called “modification localization scoring” (MS:1002491), as shown in Fig. 2. Once this term is detected in the file, the validation (and reading) software expect the following additional features to be present. First, some approaches apply a statistical threshold for accepting or rejecting that a modification position has been confidently identified, which can be reported in the <*Threshold*> element. The (re-usable) <*Peptide*> element has an attribute *via* which the residue and location of a modification can be recorded. To remain backwards compatible, we recommend that the software implementing modification scoring in mzIdentML should continue to use these attributes, populating it with the most likely modification position. A new CV term (mandatory when MS:1002491 is present in <*SpectrumIdentificationProtocol*>) must be added to every <*Modification*> element, called “modification index” (MS:1002504), where the value serves as a unique identifier (local only to the containing <*Peptide*>) to be referenced from <*SpectrumIdentificationItem*>. The modification scores (from any algorithm or scoring system) themselves can be added as CV terms with a value provided as a regular expression of four values in a defined order: *MOD_INDEX*, *SCORE*, *POSITION*, *PASS_THRESHOLD. MOD_INDEX* is a reference to the “modification index” identifier provided in the referenced <*Peptide*> − <*Modification*> element. This is required in case there are two or more different types of modification on the same peptide, which could otherwise not be distinguished by position alone. The MOD_INDEX thus ensures that the correct CV term for the modification being scored is referenced. *SCORE* is the score or statistical value for the given position. *POSITION* is the scored modification position with respect to the peptide sequence (where position = 0 is used to indicate the N terminus, and position = peptide length+1 is used to indicate the C terminus). The *POSITION* can include the bar symbol '|', as a logical OR, if the score relates to multiple positions that cannot be distinguished. *PASS_THRESHOLD* holds a Boolean (true, false) value to indicate whether the modification position passes the threshold described above. If a reader of a file wishes to determine all the sites identified without ambiguity given the threshold written to the file, one could retrieve all those PSMs with modification scores having *PASS_THRESHOLD* equals true.

Where modification position scoring, and similarly peptide-level statistics (discussed below), have been performed by post-processing software rather than the initial search engine, any relevant parameters of the post-processing should be added under <*AdditionalSearchParams*>, and the software description under <*AnalysisSoftwareList*> (not shown).

##### MS/MS cross-linking approaches

**Fig. 3.**
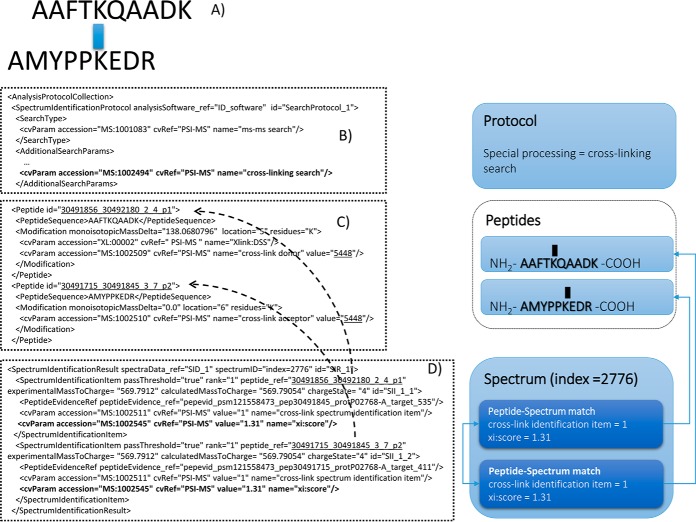
***A*, A graphical representation of the cross-linked peptide pair identified in the example.** *B*, A specific CV term is added to the header of the file to indicate that this is a cross-linking search result set. *C*, The two peptide chains identified from a given spectrum are presented in a pair of <*Peptide*> and <*Modification*> elements linked via a shared, unique value in the <*cvParam*> element. The longer peptide is flagged as the cross-link donor (carrying the mass of the cross-linking reagent) and the other peptide is flagged as the cross-link acceptor with a zero mass on the <*Modification*>. *D*, The evidence for individual identifications is captured via two <*SpectrumIdentificationItem*> elements, which may share the same score (<*cvParam*>) for the paired identification, but may also store different, individual scores for each chain identified if appropriate (not shown).

Search engines that are able to identify cross-linked peptides report PSMs in a broadly similar manner to regular search engines. However, where an identification is made indicating that a spectrum matches a cross-linked pair of peptides, there may be a score for the overall identification, as well as independent scores for the alpha (cross-link donor) and beta (cross-link acceptor) peptides. This arises because it is common for fragment products to be identified only from one or the other peptide chain, and thus a given result may include higher confidence in one peptide than in the other (37). To fulfil these requirements in mzIdentML 1.2, the following adaptations were made (Fig. 3). First, the <*SpectrumIdentificationProtocol*> must contain the CV term “cross-linking search” (MS:1002494) as shown in Fig. 3*B*. Once this term is detected in the file, the validation and implementing software will expect the following features to be present. First, a mechanism has been added for relating two different <*Peptide*> elements together, using the CV terms “cross-link donor” and “cross-link acceptor” where an identical (and within-file unique) value indicates that they are grouped together (Fig. 3*C*). The <*Modification*> element has an attribute called *monoisotopicMassDelta*, and by convention it is expected that the cross-link donor contains the complete mass delta introduced by the cross-linking reagent, and that the cross-link acceptor reports a mass shift delta of zero. As no current CV is designed for cross-linking modifications, to capture the modification masses, site specificity and common names for cross-linking reagents, we have created a new CV (XLMOD-CV) to which new terms can be added by request.

Second, a convention was also introduced within a given <*SpectrumIdentificationResult*>. There, a pair of cross-linked peptides are reported as two instances of <*SpectrumIdentificationItem*> linked together by sharing the same value for the rank attribute, and through having a shared local unique identifier as the value for the CV term “cross-link spectrum identification item” (MS:1002511), as shown in Fig. 3*D*. If the search engine has produced a single score for the cross-linked pair, both <*SpectrumIdentificationItem*> elements must carry the identical score (same CV term name and value, as in Fig. 3*D*), but the two chains may also have additional, independent scores if needed (not shown). Finally, mechanisms have also been developed that enable evidence derived from cross-linked peptides pairs containing differential stable isotope labels to be encoded, as well as protein interaction evidence (not shown, see the specification document for more details). This overall mechanism can be extended to report more than two peptides that are sequentially cross-linked. More complex scenarios are not supported in mzIdentML 1.2.

##### Peptide-level Statistics

To encode peptide-level scores or statistics in mzIdentML, first, an additional CV term “peptide-level scoring” (MS:1002490) must be included in <*SpectrumIdentificationProtocol*> (supplemental Fig. S1). Second, there are various mechanisms by which a set of PSMs can be collapsed down to a peptide-level, depending on the purpose of the routine. In contexts such as genome annotation, an application may only require evidence for whether a given peptide sequence has been confidently identified regardless of its modification status, and thus different PSMs giving evidence for both modified and unmodified forms of the peptide could be grouped together. In other cases, such as providing evidence for particular PTMs, grouping of PSMs into peptides must differentiate between modification statuses. Three CV terms have been added to the PSI-MS CV: “group PSMs by sequence” (MS:1002496), “group PSMs by sequence with modifications” (MS:1002497), and “group PSMs by sequence with modifications and charge” (MS:1002498) to cover the most common scenarios. Further CV terms for other grouping mechanisms can be added on request. One of the main reasons for performing peptide-level analysis is to apply a threshold, such as 1% FDR, for selecting data for downstream analysis, which can now be added to the search protocol. In addition, as explained, a mechanism is then needed for capturing how different PSMs are grouped into a single peptide. This is achieved by adding a CV term “peptide group ID” (MS:1002520) to every PSM (<*SpectrumIdentificationItem*>) in the file, whereby the associated value is a unique identifier shared between all PSMs in the same peptide group. In supplemental Fig. S1, the unique identifier used is the peptide sequence itself (because when grouping by sequence irrespective of the modification status, this value must be unique), although this could be any arbitrary value such as an integer code. Finally, the mzIdentML file must be able to record scores or statistical values at the peptide-level. This is achieved *via* adding CV terms with identical values to all PSMs within the same peptide-group. There are now branches within the PSI-MS CV providing different scores for PSMs and peptides from which suitable terms can be sourced. The use of peptide-level scoring and export to mzIdentML 1.2 has already been added to PeptideShaker (14) and ProteoAnnotator (19).

##### Encoding Proteogenomics Approaches

Proteogenomics data requires storing the results of mapping peptide sequences identified back onto gene models and source chromosomes potentially coming from different genome builds. This is achieved in mzIdentML 1.2 as follows. First, an additional CV term “proteogenomics search” (MS:1002635) is included in <*SpectrumIdentificationProtocol*> (supplemental Fig. S2). CV terms have been created to enable the mapping of peptides back to specific positions on chromosomes, accounting for regions where it has been inferred that a peptide is mapped across an intron boundary to different exons. CV terms related to peptide sequences (*e.g.* peptide coordinates, number of exons, etc) must be included in <*PeptideEvidence*> elements, whereas CV terms related to the gene model/resulting protein (genome build, chromosome name and strand) must be included in <*DBSequence*> elements (representing the database protein sequence), as indicated in supplemental Fig. S2.

##### Other Changes in mzIdentML 1.2

Various changes have also been made in mzIdentML 1.2 and in the accompanying implementation guidelines to better accommodate four additional common use cases: pre-fractionation of samples, approaches for *de novo* sequencing of peptides, spectral library searches and the use of multiple search engines in one combined analysis. We have also significantly improved the reporting of protein-level results, derived by protein inference, which was reported in detail here (35).

##### Prefractionation

A single mzIdentML file is intended to encompass the analysis of a single sample, either as a result of a single MS run, or as the end result of multiple MS runs from the same sample where pre-fractionation has occurred. However, to simplify the reading of mzIdentML files by software, in both mzIdentML 1.1 and continued in mzIdentML 1.2, there is a restriction that only a single list of proteins (one <*ProteinDetectionList*>) can be given in one file, although multiple <*SpectrumIdentificationList*> elements can be provided. We have amended the specification document to clarify the cases where one or many mzIdentML files are expected in cases of sample pre-fractionation. In brief, where protein inference is performed over *n* lists of PSMs (one per fraction) to produce a single protein list, this should be stored in a single mzIdentML file with *n* <*SpectrumIdentificationList*> elements and a single <*ProteinDetectionList*>. If protein inference happens independently on each fraction, then *n* mzIdentML files should be used, each containing a single <*SpectrumIdentificationList*> and one <*ProteinDetectionList*>. For a fuller discussion, consult the mzIdentML 1.2. specification document available from the PSI website.

##### Multiple Search Engines

It has been widely reported that there are gains in sensitivity for peptide and protein identification through the use of multiple search engines (38–40). In mzIdentML 1.1, it was already described how such approaches could be encoded and exported from software, but the resulting scheme was difficult to implement for reading software. The challenge arises because an mzIdentML 1.1 file could contain the search engine results as reported by the original search engines, as well as list of rescored PSMs, which were used for protein inference, and constitute the “final” results of the process. As such, in mzIdentML 1.2, we have specified that there can only be a single result for each spectrum searched (*i.e.* the spectrum identifier is unique within the file), thus enforcing that only “final” results after performing post-processing or combination can be validly reported.

##### De Novo Sequencing

There are several software packages that aim to derive complete or near complete peptide sequences directly from the spectrum without requiring a sequence database. The mzIdentML 1.1 specifications discussed that such approaches could theoretically be supported, but relevant examples files were not produced at the time because of an apparent lack of demand. It has since become evident that supporting *de novo* results was not straightforward, as there was a mandatory requirement for every PSM reported to record one to many relationships to protein sequences (see <*PeptideEvidenceRef*> on Fig. 1*A* and <*PeptideEvidence*> on Fig. 1*C*). In *de novo* approaches there is no need to relate a peptide sequence to a parent protein, and as such this cardinality has been relaxed to zero to many in mzIdentML 1.2, only when the export software includes the CV term as a “flag” in the <*SpectrumIdentificationProtocol*> “*de novo* search” (MS:1001010). In other cases, the validation software will then report an error if relationships to one or more proteins are not recorded for any PSMs.

##### Spectral Library Searching

mzIdentML 1.2 can also support searches against pre-annotated spectral libraries. The standard case for representing PSMs is modeled with scores or statistics on <*SpectrumIdentificationItem*> referencing to a <*Peptide*> element. For sequence database searches, <*Peptide*> stores the (sequence) database entry against which a spectrum has been matched. For spectral library searches, <*Peptide*> should store a representation of the spectral library entry, annotated with any metadata about the library entry (such as confidence scores or metrics for the entry itself), with or without a peptide sequence depending on what is contained within the library (*i.e.* matches against previously unidentified library entries can be supported). As for *de novo* sequencing, it is not mandatory either to provide links between peptides and database proteins (<*DBSequence*> elements) from which the peptide sequence could have been derived, because these associations may be unknown.

##### Guide on Implementing New Features in mzIdentML 1.2

In this article we describe several extensions to mzIdentML, and introduce version 1.2 from version 1.1, to support use cases that were not anticipated previously. However, aside from required minor changes to cardinality in several places (a few attributes changing to become optional or mandatory), the resulting XML schema for mzIdentML 1.2 is identical to mzIdentML 1.1. As such, we anticipate that for groups who have already implemented mzIdentML, only minor changes would be required to accommodate both mzIdentML 1.1 and 1.2 files. We expect that mzIdentML 1.1 files will remain in circulation for several years. The changes made to mzIdentML 1.2 can be described as backwards compatible, in that reading software designed for mzIdentML 1.1 should function in most cases without adaptation. However, for new implementers of mzIdentML for export from software, we strongly encourage developers to follow the mzIdentML 1.2 guidelines.

In the case of cross-linking, this is still a relatively specialized field, and thus it would not be expected for general reading software to be able to handle the extensions described beyond general reading of PSMs now. Peptide-level statistics and modification location scoring are becoming more prevalent in proteome informatics, and thus we strongly encourage development teams to support these features for both file reading and writing.

## CONCLUSIONS

The mzIdentML standard for peptide and protein identification data has been stable for around five years, and has steadily grown in use to support data interchange between software tools, as well as a data repository submission format. Here we report updates to the standard to enhance its support and usability for unanticipated requirements when the standard was initially released. We have attempted to encode these use cases without adapting the core model of the format to simplify adoption by the developer community. The PSI remains a free and open consortium of interested parties, and we encourage critical feedback, suggestions and contributions via attendance at a PSI annual meeting, conference calls or our mailing lists (see http://www.psidev.info/).

## Supplementary Material

Supplemental Data

## Footnotes

^1^ The abbreviations used are:

PSI: Proteomics Standards Initiative
CV: controlled vocabulary
FDR: false discovery rate
PIA: protein inference algorithms
PSM: peptide spectrum match
PTM: post-translational modification
XML: Extensible Markup Language
XSD: XML Schema Definition.
