# Supplementary material for: The mzIdentML Data Standard Version 1.2, Supporting Advances in Proteome Informatics
Source: Mol Cell Proteomics. 2017 May 17;16(7):1275–85. doi: 10.1074/mcp.M117.068429 (PMC5500760; doi:10.1074/mcp.M117.068429)
Supplement: Supplemental Data [file supp_16_7_1275__index.html]

The mzIdentML data standard version 1.2, supporting advances in proteome informatics — The mzIdentML Data Standard Version 1.2, Supporting Advances in Proteome Informatics — mzIdentML Data Standard Version 1.2 — Supplemental Data 

# The mzIdentML Data Standard Version 1.2, Supporting Advances in Proteome Informatics

## Supplemental Data

- Supplementary Figures (.pdf, 673 KB) - Supplementary Figures showing additional encodings of special cases in mzIdentML 1.2
