# Supplementary material for: The mzIdentML Data Standard Version 1.2, Supporting Advances in Proteome Informatics
Source: Mol Cell Proteomics. 2017 May 17;16(7):1275–85. doi: 10.1074/mcp.M117.068429 (PMC5500760; doi:10.1074/mcp.M117.068429)
Supplement: Supplemental Data [file 10.1074_M117.068429_mcp.M117.068429-1.pdf]

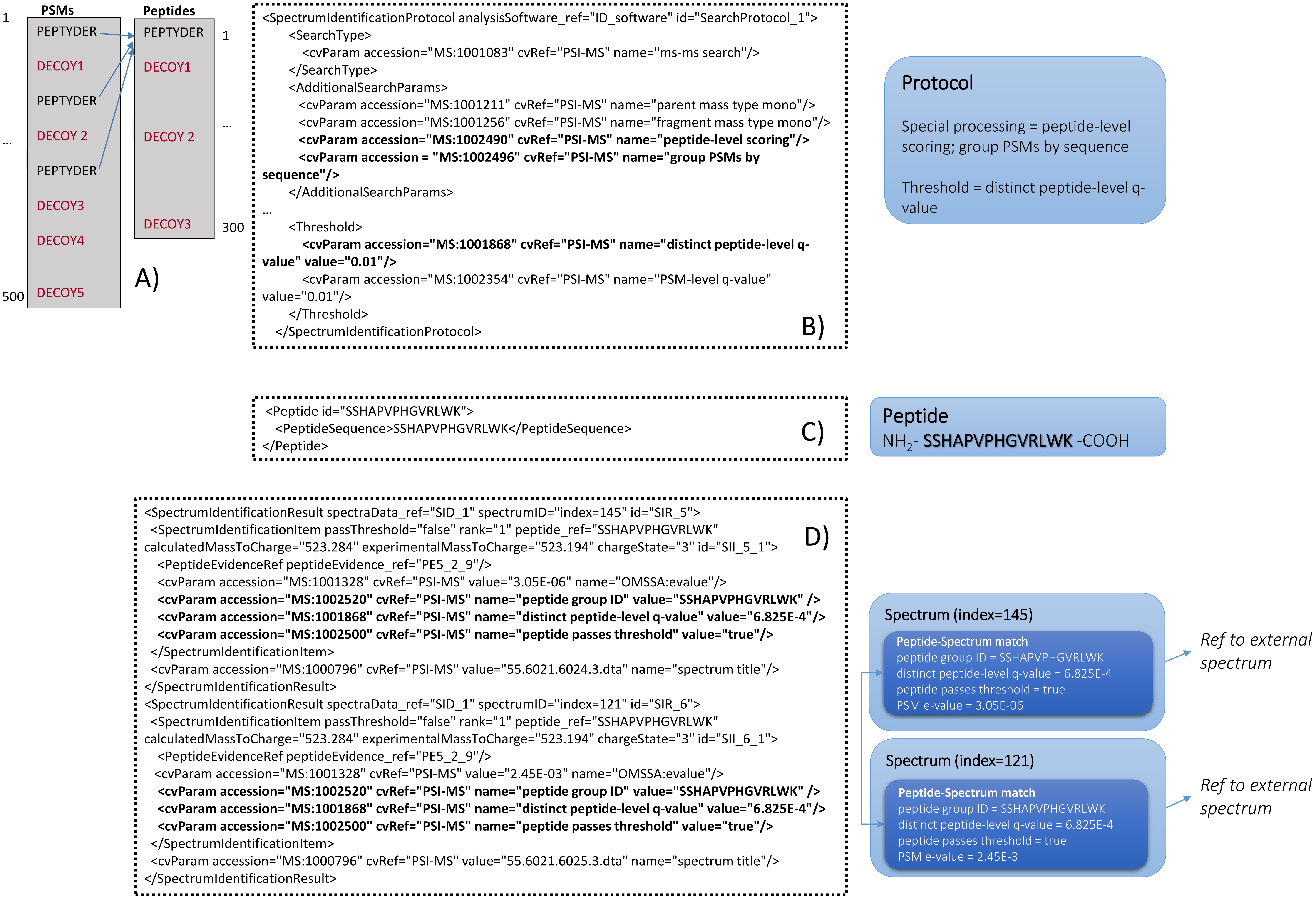

Suppl. Figure 1. A) A graphical representation of the process by which redundant PSMs identifying the same peptide can be collapsed down into a peptide list and re-scored. Decoys are often identified by a single PSM only, and thus peptide-level FDR estimation tends to be more conservative than PSM-level FDR estimation. B) The search protocol must include a CV term to indicate that peptide-level re-scoring has been encoded, and the threshold implemented in the file for accepting or rejecting peptide identifications. C) Different PSMS reference to the same re-usable *<Peptide>* element stored in the file D) Peptide-level scores are represented in *<SpectrumIdentificationItem>* (SII) by grouping different SIIs with a shared value for "peptide group ID", shared scores and shared values for "peptide passes threshold".

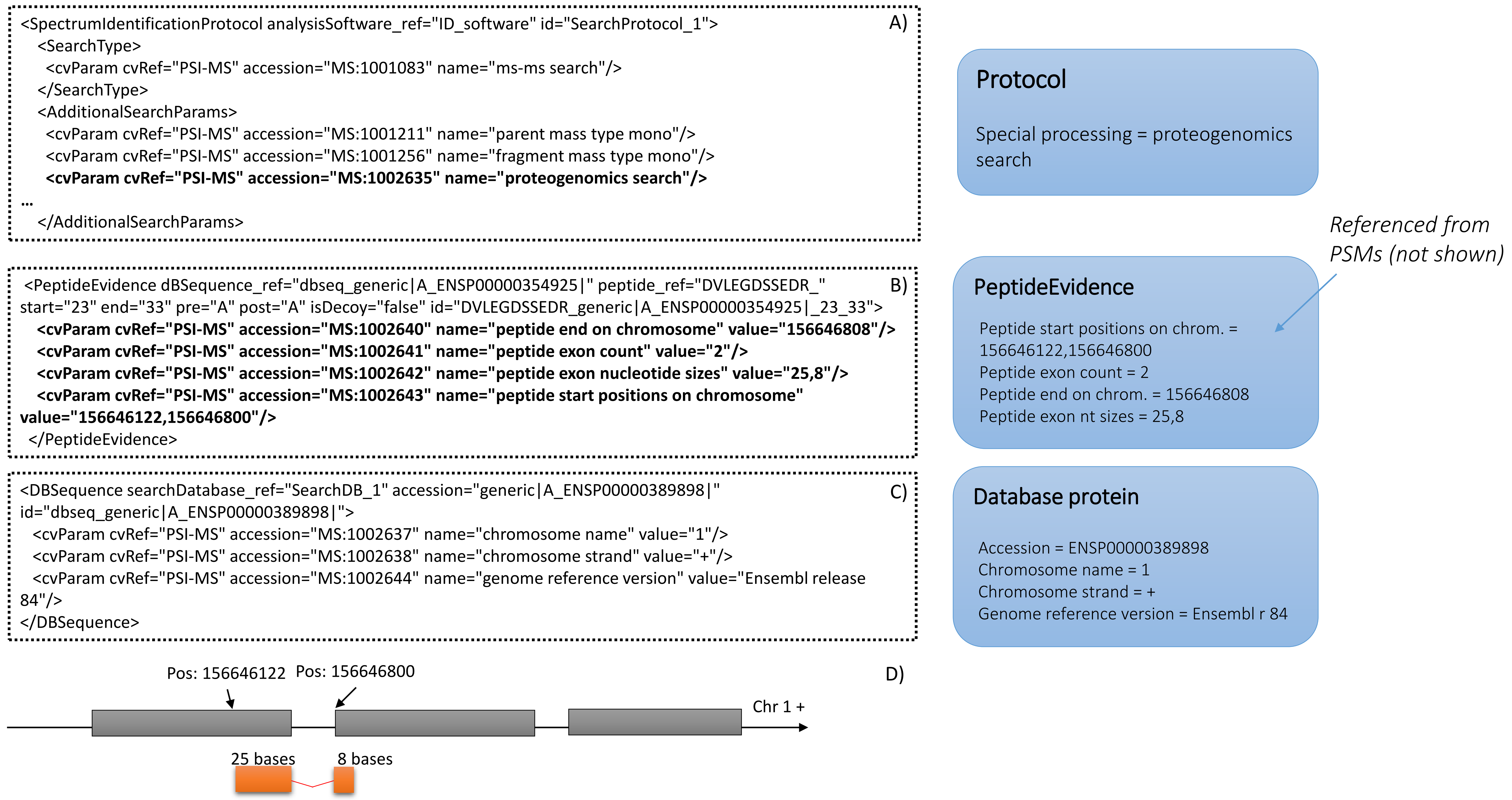

Suppl. Figure 2. A) If a proteogenomics approach has been followed, there is a mandatory CV term inserted into the protocol. B) There are four mandatory attributes encoding the mapping of the peptide onto the chromosome, according to a particular protein/gene product in which it can be identified. C) The chromosome strand, number/name and genome reference version are stored on the database protein record. D) A visualization of an example mapped intron-spanning peptide (in orange) onto exons is indicated in grey.
